# Observation resolution critically influences movement-based foraging indices

**DOI:** 10.1038/s41598-019-50017-2

**Published:** 2019-09-20

**Authors:** Michael Kalyuzhny, Tom Haran, Dror Hawlena

**Affiliations:** 1 Department of Ecology, Evolution & Behavior, Alexander Silberman Institute of life Sciences, The Hebrew University of Jerusalem, Edmond J. Safra Campus, Jerusalem, 91904 Israel; 2 Herpetological Collection, National Natural History Collections, The Hebrew University of Jerusalem, Jerusalem, Israel

**Keywords:** Behavioural ecology, Ecological modelling

## Abstract

Movement‐based indices such as moves per minute (MPM) and proportion time moving (PTM) are common methodologies to quantify foraging behavior. Hundreds of studies have reported these indices, many without specifying the temporal resolution of their original data, and others using varying resolutions. This was done despite the likelihood that observation resolution can affect MPM and PTM estimates. Our goal was to empirically determine the sensitivity of these foraging indices to changes in the temporal resolution of the observation. We used a high-speed camera to record movement sequences of 20 *Acanthodactylus boskianus* lizards. Then, we gradually decreased the resolution of the data and calculated the foraging indices at different temporal resolutions. When considering the range of temporal resolutions that are relevant for field observations with unassisted vision, we found 68% and 48% difference in MPM and PTM estimates, respectively. When using the highest resolution, our estimate of MPM was an order of magnitude higher than all prior reported values for lizards. Our results raise major concerns regarding the use of already published movement-based indices, and enable us to recommend how new foraging data should be collected.

## Introduction

The study of animal foraging behavior has been a key topic in ecology and evolutionary biology^1,2^. Comparison of foraging behavior across species and in conjunction with other traits requires condensing the myriad of searching, pursuit, and capture behaviors to simple and comparable quantitative indices^2^. This is probably why simple movement-based indices, and especially Moves Per Minute (MPM) and Proportion Time Moving (PTM), have become very popular, and are still being utilized extensively to characterized foraging behavior across taxa (e.g. Lizards: Reilly *et al*., Halperin *et al*.^2,3^; Fish: Fu *et al*., Radabaugh, Davis *et al*.^4–6^; Snakes: Hansknecht and Burghardt^7^; Insects: Ferris and Rudolf, Mundahl and Mundahl^8,9^). For example, the foraging behavior of approximately 170 lizard species has been characterized using PTM and MPM (see Halperin *et al*.^10^ for a detailed account).

Huey & Pianka^11^ were the first to introduce PTM and MPM. They recorded the “duration of each move and duration of each stop” of seven Kalahari lizard species, and divided the cumulative duration of all movements or the number of discrete movements by the total observation duration for estimating PTM and MPM, respectively. The authors did not explain how move and stop were defined, and did not specify the minimal durations of the moves and stops they used (*M*_*min*_ and *S*_*min*_, respectively). This potentially important information has not been explicitly reported also in many succeeding studies, and those that did report it vary in the duration of *M*_*min*_ and *S*_*min*_ and the ways these movement and stop bouts were determined. For instance, Verwaijen and Van Damme^12^ considered pauses of one or more seconds as bouts of immobility, while Plessis & Mouton^13^, and Cooper and Whiting^14^ did not record bouts of immobility that were shorter than two seconds. Similarly, Sales & Freire^15^ defined the lizard as static only after counting one second mentally after the lizard stopped moving, and Hawlena *et al*.^16^ did not record movements that were shorter than 3 seconds.

Recently, Halperin *et al*.^10^ raised a concern that variation in *M*_*min*_ and *S*_*min*_ may seriously affect MPM and PTM estimates. They suggested that different *M*_*min*_ and *S*_*min*_ used to characterize exactly the same movement sequence could yield different MPM estimates. This is because as *M*_*min*_ and *S*_*min*_ decrease, more movement events are included in the calculation, increasing MPM. This problem is expected to affect also the PTM calculation because as *S*_*min*_ increases, fewer stop events are included in the calculation, increasing the cumulative time spent moving. Furthermore, inconsistent determination of *M*_*min*_ and *S*_*min*_ both within and between studies may inflate the inaccuracy and imprecision of the MPM and PTM estimates, possibly affecting case studies and comparative studies alike. An important next step is thus to empirically characterize the degree to which movement-based indices are dependent upon *M*_*min*_ and *S*_*min*_.

To address this challenge, we recorded the foraging behavior of 20 Bosc’s fringe-toed lizards (*Acanthodactylus boskianus* Daudin 1802) in the field, using high speed imagining (120 frames per second). After digitizing the data, we gradually degraded the observation resolution using three schemes that may reflect different field observation practices that relay on unassisted vision. We used the manipulated data to conduct a sensitivity analysis that examines to what extent estimates of PTM and movement frequency rely on *M*_*min*_ and *S*_*min*_. To characterize the movement frequency, we followed the recommendation of Halperin *et al*.^10^, and used Changes Per Minute (CPM) instead of the classic MPM. This frequency index is very similar to MPM but does not suffer from inherent bias and inflated error that are typical of MPM (see Halperin *et al*.^10^ for details). CPM is calculated by dividing the number of initiations of stops and moves by the total observation duration (minus one time unit, since no changes can be observed after it). Thus, CPM/2 approximately equals MPM, making these indices fully comparable.

A possible way to reduce variation in the determination of *M*_*min*_ and *S*_*min*_ is to record the animal behavior more precisely, and to calculate the indices using the shortest movement and stop durations. Yet, this approach may artificially inflate PTM and CPM estimates. This is because animal locomotion is likely a product of several biological processes; hence not all moves and stops are necessarily relevant for quantifying foraging behavior. In other words, the relevant minimal move and stop durations may be higher than the absolute *M*_*min*_ and *S*_*min*_ values. Consequently, our second goal was to search for statistically sound and biologically relevant criteria for determining *M*_*min*_ and *S*_*min*_.

## Methods

### Study site and species

We observed 7 adult and 13 hatchling Bosc’s fringe-toed lizards *(Acanthodactylus boskianus*) in three field sites in the central Negev desert, Israel (30^0^42′N 34^0^46′E). All sites were characterized by flat fluvial beds scattered with small perennial shrubs, mostly *Hammada scoparia* and *Artemisia sieberi*. A previous study estimated that *A. boskianus* adults have an MPM value of 2.66 and a PTM value of 0.39, and hatchlings have an MPM value of 2.15 and a PTM value of 0.29^17^.

### Data collection and digitization

We observed lizards’ foraging behavior in the field during September 2016 and August 2017, when adult and hatchling lizards co-occurred. All observations were conducted by the same observer (TH) between 08:30 and 12:00 AM and between 15:00 and 17:30. Lizards were located by random search. Limiting our observations to these peak activity times is concordant with commonly used protocols for measuring foraging behavior, ensuring that thermoregulation behavior does not confound our foraging measurements (Perry, 2007). When a lizard was spotted, the observer started recording its behavior at 120 frames per second, using a Panasonic HC-W850 camcorder, mounted on a tripod. While recording, the observer stood motionless and kept a distance of 3–5 meters from the lizard. If the lizard moved out of sight, the observer slowly moved the camera to a better spot, keeping the stream of recording. All observations were 20–25 minutes long. To minimize consequences of confounding factors on our foraging sequences, and to make our data comparable to previous studies, we ignored observations in which lizards were hidden for almost the entire observation period, engaged in social interactions (e.g., chasing conspecifics), or thermoregulating (e.g., staying motionless for long periods in the shade). We also deleted the first three minutes of every observation to discard the habituation time of both lizard and observer.

We used the Behavioral Observation Research Interactive Software (BORIS^18^) to digitize the behavioral data. Videos were replayed on the computer at low speed and the time of beginning and ending of each event was recorded. We defined moving events as any relocation of the lizard’s center of mass. Thus, during a stop event a lizard is not necessarily motionless, and can handle a food item or wave its arms. We also recorded the beginning and ending of periods in which the lizard was hidden, i.e. out of frame or not visible due to a visual obstruction.

### Data analysis

To study the effect of *M*_*min*_ and *S*_*min*_ on PTM and CPM estimates, we artificially reduced the resolution of the original data so that short stops and/or moves were not used for their calculation. To simplify the analysis, we used a single parameter for the temporal resolution of the artificial data, denoted by *τ*. The way short moves and stops are being recorded can differ dramatically within and between studies. This is because the observers may vary in skills and recording methods. Since different studies measured short stops and moves differently, we simulated different scenarios using three different schemes of “data degradation”. Based on our personal field experience, we assumed that it is easier to notice and record short moves than short stops. Thus, in all schemes, stops shorter than *τ* were “missed” and incorporated into adjacent moves, but short moves were treated differently using three schemes:

- leaving short moves unchanged. This scheme assumes that the observer can record even short moves but not short stops. This scheme is simplistic, but it allows directly relating the dependence of CPM on *τ* to the distribution of stop durations (more on that below).
- omitting all moves shorter than *τ*. This scheme assumes the observer deliberately ignored or missed short moves.
- lengthening moves shorter than *τ* into length *τ*. The elongated moves begin at the same time as the original move but end later. Since this elongation can generate new stops shorter than *τ*, any stop that is now shorter than *τ* is transformed into movement time. This process of transforming stops into moves and elongating moves is repeated until the movement sequence does not change any more, which happened when there are no moves or stops shorter than *τ*. This scheme assumes that short movements are easy to detect, but take more time to record.

All three schemes were applied to the 20 individuals in *τ* intervals of 0.01 seconds, and PTM and CPM were calculated for each output sequence and plotted against *τ*. Periods when the individual was hidden were removed from the total observation time, and we did not consider any possible changes (initiation or end of movement) that could occur in that period.

It is interesting to note that a visual inspection of the dependence of CPM on *τ* can give us important information on the nature of the distribution of stop durations. As *τ* is increased, more and more stops are discarded and moves are merged, reducing CPM by the proportion of stops that are shorter than *τ*. Hence, the dependence of CPM on *τ* is simply:

1 $${\rm{CPM}}(\tau )\approx {{\rm{CPM}}}_{0}(1-{{\rm{F}}}_{{\rm{t}}=\tau }),$$

where CPM_0_ is the CPM when no stops are discarded and F_t=τ_ is the cumulative distribution function of the stop durations evaluated at *τ*. The formula above is not exact mostly because of missing periods in the data, and is true only for scheme a.

Animal locomotion is likely a result of several biological processes, suggesting that not all stops are clearly relevant for quantifying foraging behaviors. Consequently, our second goal was to search for statistically sound criteria for determining the appropriate resolution. We used the bout criterion^19^, denoted here by θ^*^, to separate stop intervals within bouts of movement, from stop intervals between them. The classical approach for identifying bout criteria is to separate the distribution of stop durations into components, often by fitting a mixed distribution (e.g., using log-survivorship analysis^20^; log-likelihood analysis of mixed exponential distributions^21^ or log-transformation of the intervals^22^ and fitting a mixture of gaussians). We attempted a slightly different approach. In line with the suggestion of Clauset *et al*.^23^, we fitted (using maximum likelihood methods) only the long stops, above a cut-off duration *θ*, to several candidate distributions – Pareto (power law), Weibull, gamma and lognormal. Stops shorter than θ were thus discarded. To find the cut-off point, we fitted each distribution over a range of *θ*s, from 0.01 seconds to 3 seconds in intervals of 0.01 seconds. For each fit we then calculated the Kolmogorov-Smirnov (K-S) goodness of fit statistic. If the long moves (above θ^*^) indeed resemble one of these distributions and the short ones do not, we would expect, as suggested by Clauset *et al*.^23^, that as *θ* increases, the K-S values would rapidly decrease to a minimum around θ^*^ and then slowly increase. This is because the very short moves belong to a different distribution, and removing them from the fit by increasing the threshold *θ* strongly improves the goodness of fit. Increasing *θ* further, beyond θ^*^, results in a decrease of sample size, reducing goodness of fit and increasing K-S. Hence, the *θ* corresponding to the minimal K-S value can be regarded as an estimator of θ^*^. This optimization procedure was performed for all four candidate distributions, and we chose the θ^*^ of the distribution with the lowest value of K-S.

Our estimate of θ^*^ cannot be reliable if the fit of the distribution is poor. To test distribution fit, we fitted for each individual the four distributions using θ^*^, compared their relative goodness of fit using AIC weights and tested whether they are plausible models using a K-S test. Finally, the above analyses assume that stops are independent. To test this assumption, we tested the lag 1 autocorrelation of stop durations. All analyses were performed using Matlab 2016a.

## Results

**Figure 1:**
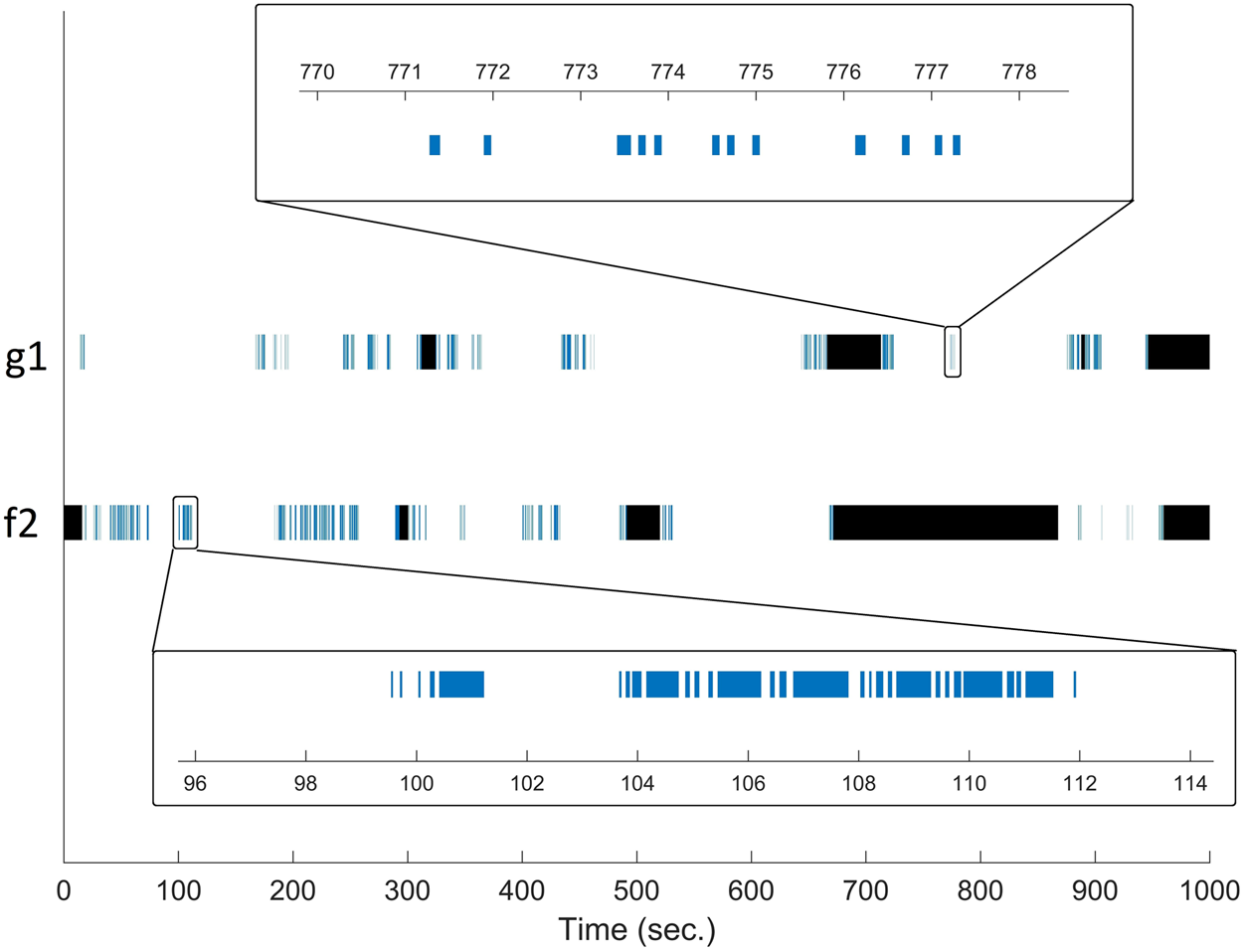
Representative movement patterns of two *A. boskianus* lizards (coded “f2” and “g1”). The movement periods (blue), stopping periods (white) and periods when the animal was hidden (black) are presented for 1000 seconds. We also focus on two short time periods, one with few moves and one with more moves. It is evident that there is a very wide distribution of stops; most are very short while some last >100 sec. On the other hand, there are no long continuous moves.

*Acanthodactylus boskianus* were motionless most of the observation time, at times for very long periods (>100 sec.). Their movements were highly fragmented by many frequent short stops (Fig. 1). Considering all individuals, the longest continuous movement recorded was 2.9 sec., and only 0.12% of movements were >2 sec. The longest stop that was recorded was 532 sec., while 63% of stops were <0.2 sec., and 81% of the stops were <0.5 sec. This behavior led to very high values of CPM - the mean over lizards was 57.9 ± 4.7 (SE). There were very few (<0.2%) short moves and stops with duration on the order of a single frame, hence, the true *S*_*min*_ and *M*_*min*_ are likely close to 1/120 seconds. Moreover, no difference in PTM (t test, P = 0.388) or CPM (t test, P = 0.115) was found between juvenile and adult lizards.

**Figure 2:**
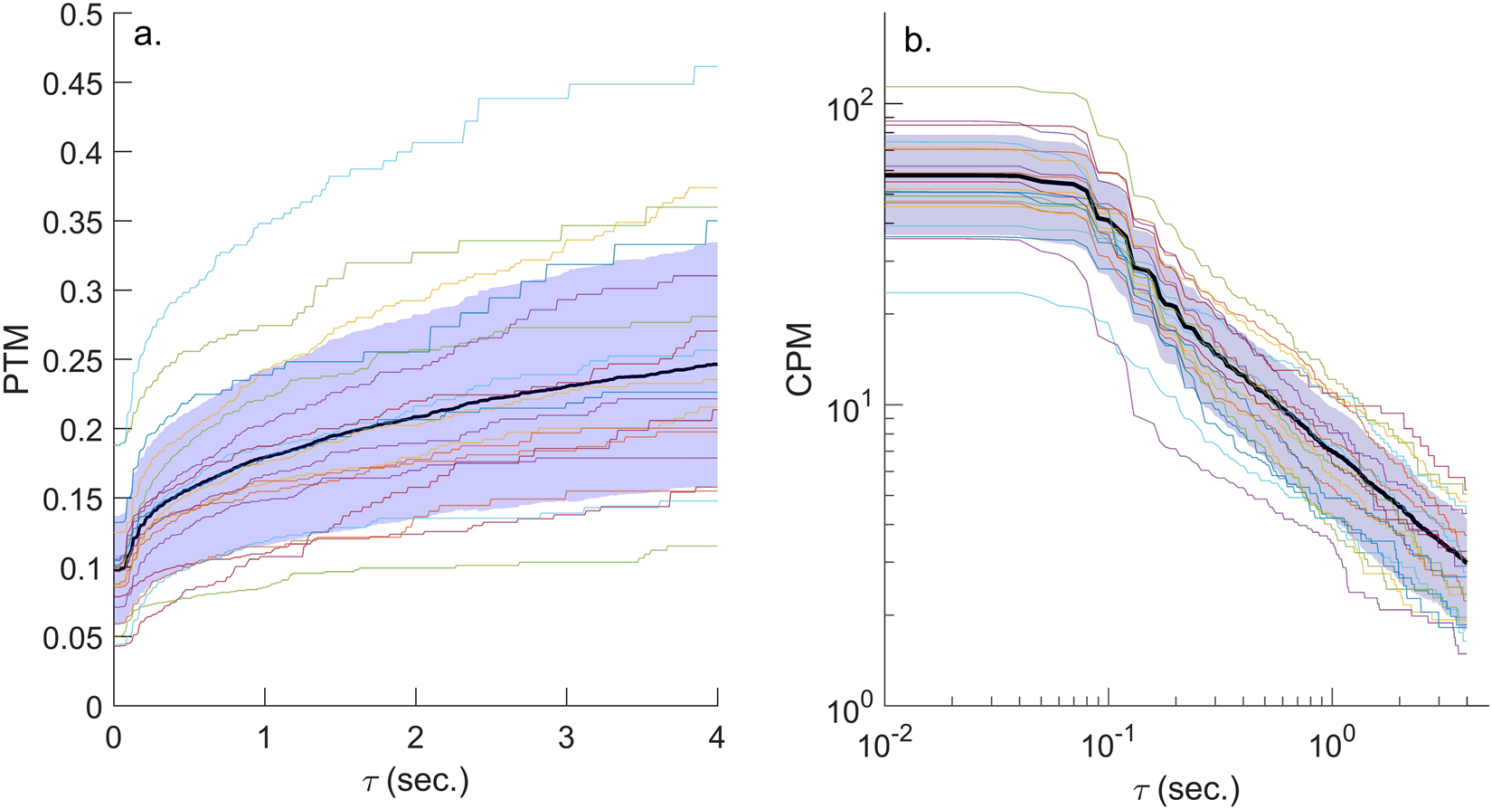
Dependence of foraging indices on the minimal durations of observed stops, *τ*, using scheme a. Starting with the original movement sequence of the 20 *A. boskianus* individuals, all stops shorter than *τ* were not observed and were instead transformed into movement time, and PTM (**a**) and CPM (**b**) were calculated for the new sequences. Each colored curve represents one individual, while the black line is the average and the lilac-colored region is 1 SD around this average. Note the double logarithmic scale of b.

We found that, regardless of the methodology used to degrade the resolution of the original data, both CPM and PTM had a strong dependence on *τ*. Figure 2 presents the simple case when we transformed stops shorter than *τ* into moves and left the moves shorter than *τ* unchanged (scheme a). In this case, when *τ* increased from 0.5 sec. to 3 sec. (which is approximately the relevant range for *τ* in field studies), average PTM increased from 0.156 to 0.231 (a 48% increase), and an increase from 1 sec. to 3 sec. resulted in a 29% increase in PTM (Fig. 2a). The effect on CPM was even more dramatic: an increase in *τ* from 0.5 sec. and 1 sec. to 3 sec. resulted in a decrease in average CPM from 10.91 to 3.51 (by 68%) and from 6.97 to 3.51 (by 50%), respectively (Fig. 2b). We found similar trends when applying the other two data degradation schemes (supplementary Figs S1 and S2).

Figure 2b hints that the distribution of stops is wide (possibly a power-law), because the dependence of CPM on *τ* seems linear on a double logarithmic axis (see Eq. 1). This seems true above some threshold of stop duration, because for short stops this straight line turns into a different shape. This was corroborated by our analysis of the distribution of stop durations. The average value of the crossover θ^*^ for all lizards was 0.35 ± 0.13 sec. (see supplementary Fig. S3 for the search results and Supplementary Table S1 for the values). The search for the crossover θ^*^ at the individual level was not very reliable due to the limited number of stops each lizard performed. The lognormal and power-law distributions were indeed plausible distributions for the stop durations: their deviation from the empirical distribution of stops was significant in zero and two individuals (α = 0.05), respectively. Furthermore, in 18 of the 20 lizards, one (or both) of these distributions was much more plausible than the other candidates, considering AIC weights (Table S1).

The lag 1 autocorrelation of stop durations was not significantly different than zero for all 20 individuals, justifying our treatment of stop events as independent.

## Discussion

We have empirically shown that the two most popular movement-based indices for quantifying foraging behavior, CPM (or MPM) and PTM are sensitive to the determination of the minimal duration of moves and stops. This result holds even when considering just the range of minimal durations that are relevant for field studies using unassisted vision (~0.5–3 sec.). When using the highest resolution, our estimate of CPM was an order of magnitude higher than all prior reported values for lizards. We have also uncovered a crossover point around a minimal stop duration of 0.35 sec. that may reveal the relevant resolution for quantifying the foraging behavior of *A. boskianus*.

Qualitatively, the dependency of PTM and CPM on the minimal duration of moves and stops is rather trivial. If short moves and stops are missed then MPM must be smaller, and when short stops are not observed or short moves are recorded as longer moves, PTM must increase. Yet, we were genuinely surprised by the magnitude of this effect. When comparing the highest resolution to a 1 sec. resolution the estimate of PTM increased by 83% and the estimate of CPM decreased by 88% (considering scheme a). When using the highest resolution, our estimate of CPM (averaged over the individuals and equivalent to MPM≈28.9) was an order of magnitude greater than the highest MPM estimate for lizards reported thus far (i.e., *Psammodromus hispanicus, MPM* = *4.71*^24^; *see* Halperin *et al*.^10^ for comparison).

We attributed this unexpected result to the distribution of stop durations. We found that above a threshold of approximately 0.35 sec., the stop durations are well-described by a single, heavy tailed distribution with high kurtosis, resembling a lognormal or a Power law distribution. In such distributions, the vast majority (and the mode) of the stops are very short, but longer stops still occur, and there is no typical time scale above which stops become rare (in the range 0.35–4 sec.). Hence, when *τ* is increased, many very short stops are transformed into movement time, considerably increasing PTM. Longer stops are still quite common, and therefore we observe a steady increase in PTM with *τ*. The strong decrease in CPM while increasing *τ* occurs for similar reasons. The high frequency of short stops leads, when they are ignored, to a decrease in CPM, and because there are also a considerable number of longer stops, this decrease is not limited to only a small range of *τ*.

It is impossible in retrospect to assess what range of minimal stop duration values has been used for calculating MPM and PTM. Based on our field experience and on the published *S*_*min*_ durations, we assumed that a range of minimal stop durations of 0.5–3 sec. should encapsulate the realistic variation used in previous field studies. We found a 48% difference in PTM and 68% in CPM estimations when comparing those two *S*_*min*_ values. If our results are representative at least of species with intermittent locomotion, then the variation in the determination of *S*_*min*_ and *M*_*min*_ both within and between studies should inflate inaccuracy and imprecision in the published CPM and PTM estimates. These large biases, which are on the order of the measured values, could seriously impair analyses and conclusions that are based on these data. Moreover, such unaccounted source of variation may seriously question the way these indices have been used and interpreted in dozens of comparative studies during the last 3 decades^2,3,25^. For example, several investigators have provided empirically derived boundaries for classifying lizard foraging modes. Cooper and Whiting^26^ found that sit-and-wait foragers exhibited PTM < 0.15 while lizards above this cut-off are active foragers. Our results (using scheme a) showed that when *S*_*min*_ increased from 0 sec. (no change in raw data) to 0.35 sec. and to 2 sec., the PTM value increased from 0.0978 to 0.1475 and to 0.2087, respectively. Hence, changes in *τ* could qualitatively alter the foraging mode classification of this species.

The strong dependence of CPM and PTM on *τ* essentially raises the question: what value of *τ* should be used to quantify foraging behavior? The distribution of stop durations can be described as a mixture of a lognormal or power law above a crossover θ^*^ and by a considerably different distribution below θ^*^. A reasonable explanation for these findings is that stop events below and above θ^*^ are generated by two different processes. Using the common interpretation of bout criteria^21,22^, we assumed that θ^*^ distinguishes between longer stops that separate move bouts and short stops that happen within a movement bout, and may not be informative for quantifying foraging behavior. We instead suggest that short stops under θ^*^ that occur within a longer movement bout may serve a different function. Breaking long movement bouts with many very short stops may cause motion dazzle that, together with the longitudinal dorsal stripes of *A. Boskianus*, may impair the predator’s ability to assess the lizard’s velocity, and consequently to intercept it^3^. Yet even if one interprets the meaning of this threshold value differently, our findings suggest that setting the minimal stop value arbitrarily above this threshold has no statistical justification. Hence, we suggest discarding stops below the crossover θ^*^ and taking into consideration all moves and stops above it. Of course, the value of θ^*^ should be evaluated case-by case.

To encourage better use of movement-based indices for quantifying foraging behavior we next discuss the use of already published data, and recommend a new protocol for collecting and calculating PTM and CPM in the field. Published data should be treated with great caution because the published values heavily rely on the arbitrary ways in which *S*_*min*_ and *M*_*min*_ have been determined. This problem is likely more severe for species with intermittent mobility that exhibit short and frequent moves and stops. Species with low mobility are less likely to perform short and frequent moves and stops because this behavior may interfere with their sit-and wait strategy. Thus, published PTM (and even CPM) estimates for such species are expected to be more reliable. Highly mobile species may use multiple short stops, but their effect on PTM (relative to mean PTM) is expected to be less pronounced because their PTM is high anyway and transforming some short stops into additional movement time most likely would not create a strong effect. Generally, published data can be trusted if there is a compelling reason to believe that the animal does not perform frequent short stops. Short field observations may assist determining this point.

When new data is collected we recommend recording the foraging behavior with a high-speed camera, encoding the movies after clearly defining movement, and then analyzing the distribution of stops in order to find the bout criterion above which all stops are clearly relevant for quantifying foraging. Finally, we suggest calculating PTM and CPM based on all moves and stops that are greater than the bout criterion, and reporting the details of this procedure to enhance comparability and reproducibility.

In summary, movement‐based indices such as PTM and MPM are common methodologies to quantify foraging behavior that are highly advantageous for comparative evolutionary‐ecological studies. Our work suggest that the ways foraging data have been collected, analyzed and interpreted in hundreds of studies is potentially flawed. By precisely recording the foraging behavior of *A.boskianus* lizards and artificially reducing the resolution of these observations, we showed that in this lizard the determination of the minimal stop and move duration substantially affect the PTM and CPM estimates. If these findings hold for other species, then the use of published PTM and MPM estimates in comparative studies must be treated with extra caution. Nevertheless, we believe that with the lack of better alternatives, the use of simple and informative statistics such as PTM and CPM for comparative studies will persist in the foreseen future. Thus, we hope that our proposed procedure for identifying the minimal move and stop duration, together with the recent recommendations of how to use (and not to use) movement-based foraging indices^10^, will add rigor to the way foraging data is being collected, analyzed and used.

## Supplementary information

Supplementary information for Observation resolution critically influences movement-based foraging indices

## Data Availability

All foraging data and the Matlab code used in the analysis are available at 10.6084/m9.figshare.9792722.v1.
