# Supplementary material for: Observation resolution critically influences movement-based foraging indices
Source: Sci Rep. 2019 Sep 20;9:13636. doi: 10.1038/s41598-019-50017-2 (PMC6754423; doi:10.1038/s41598-019-50017-2)
Supplement: Supplementary file 1 — Supplementary information for Observation resolution critically influences movement-based foraging indices [file 41598_2019_50017_MOESM1_ESM.docx]

Supplementary information for

Observation resolution critically influences movement-based foraging indices

Michael Kalyuzhny^1,a^, Tom Haran^1,2^ and Dror Hawlena^1,2^

^1^ Department of Ecology, Evolution & Behavior, Alexander Silberman Institute of life Sciences, the Hebrew University of Jerusalem, Edmond J. Safra Campus, Jerusalem **91904**, Israel.

^2^ Herpetological Collection, National Natural History Collections, the Hebrew University of Jerusalem, Jerusalem, Israel.

^a^ Corresponding author: michael.kalyuzhny@mail.huji.ac.il


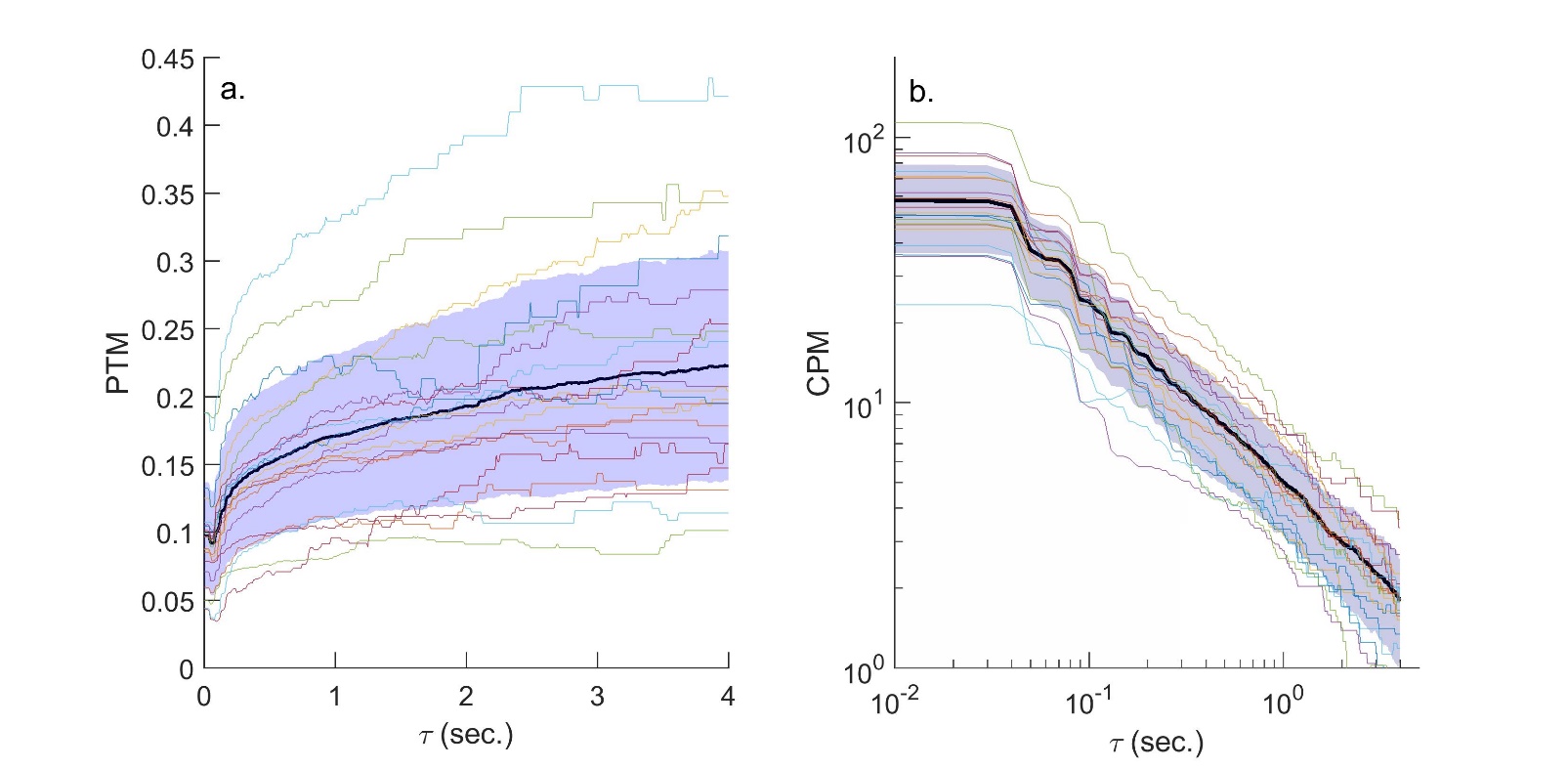


Fig S1 – Dependence of foraging indices on the minimal durations of observed stops and moves, *𝜏*, using scheme b. Starting with the original movement sequences of the 20 *A. boskianus* individuals, all stops shorter than *𝜏* were not observed and were instead transformed into movement time, and following that all moves shorter than *𝜏* were not observed and were instead transformed into stopping time. PTM (**a**) and CPM (**b**) were calculated for the new sequences. Each colored curve represents one individual, while the black line is the average and the blue region is 1 SD around this average. Note the double logarithmic scale of b.


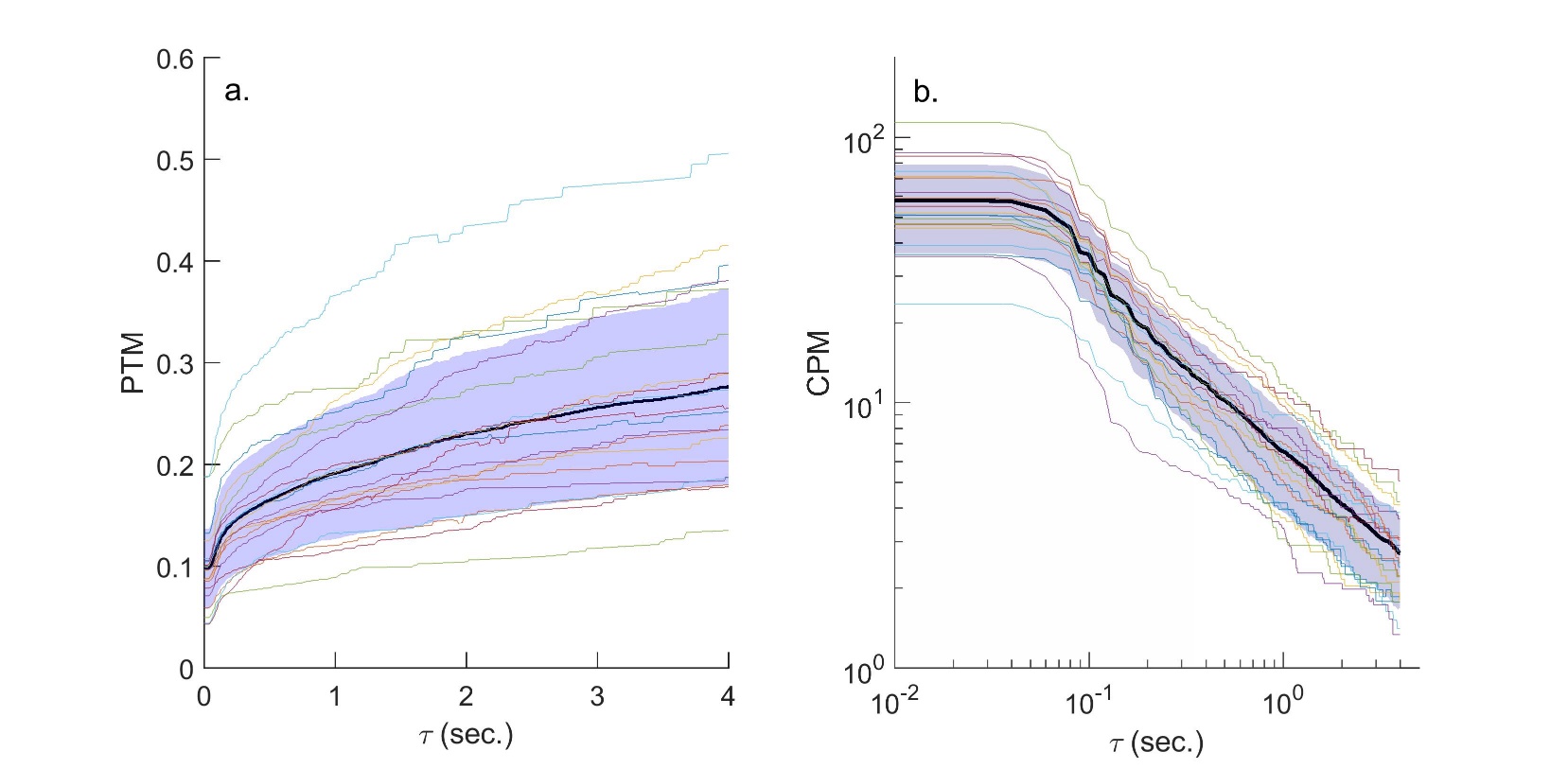


Fig S2 – Dependence of foraging indices on the minimal durations of observed stops and moves, *𝜏*, using scheme c. Starting with the original movement sequence of the 20 *A. boskianus* individuals, all stops shorter than 𝜏 were not observed and were instead transformed into movement time, and following that all moves shorter than *𝜏* were elongated forward in time and transformed into moves of time *𝜏*. These two steps were repeated until the sequence did not change further. PTM (**a**) and CPM (**b**) were then calculated for the new sequences. Each colored curve represents one individual, while the black line is the average and the blue region is 1 SD around this average. Note the double logarithmic scale of b.


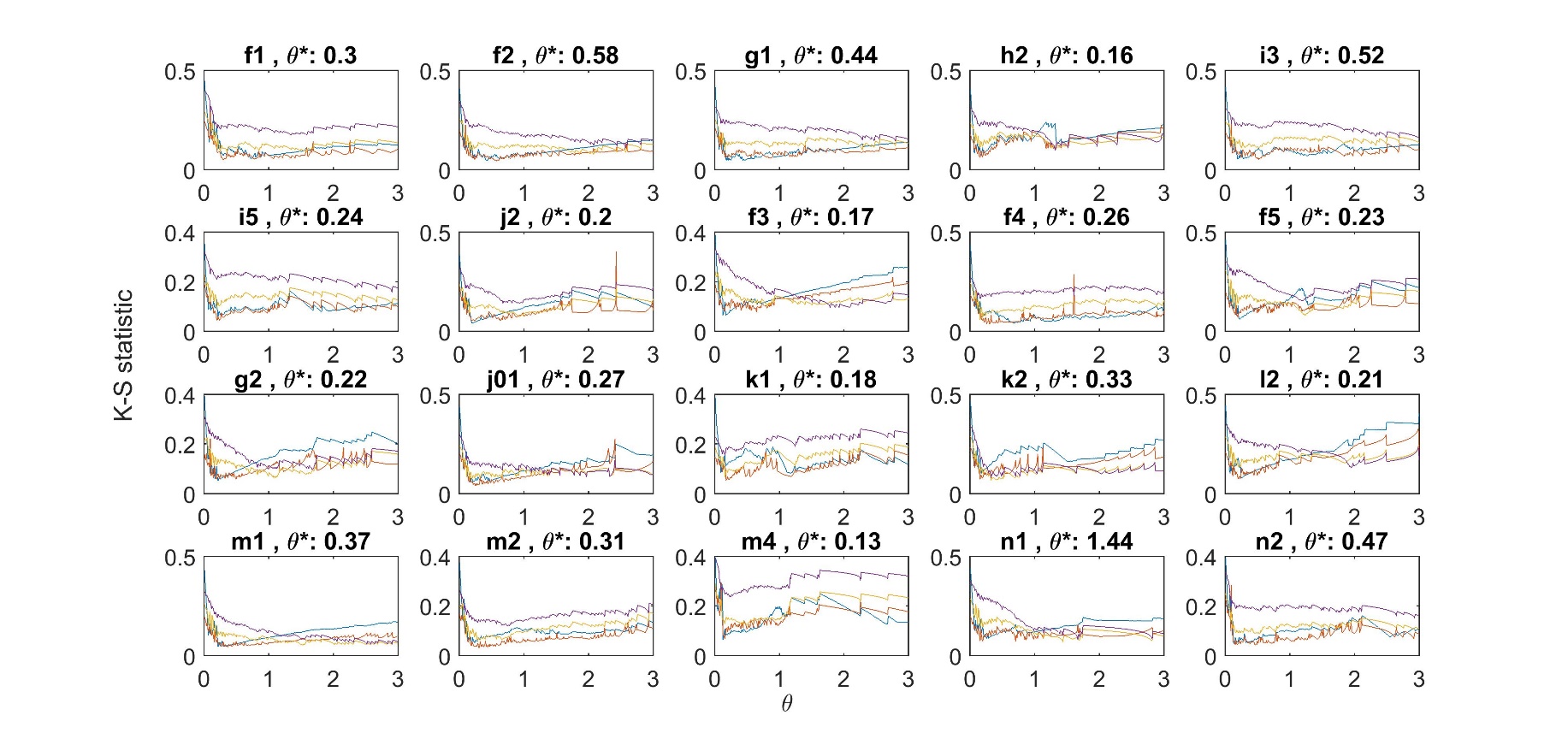


Fig. S3 - Estimation of the crossover (*θ**) between short and long stops in the distribution of stop durations for 20 *A. boskianus* individuals. For values of the crossover parameter *θ* between 0 sec. and 3 sec., all stops shorter than *θ* were discarded, and the other stops were fitted using maximum likelihood to the pareto (blue), lognormal (red), weibull (orange) and gamma (purple) distributions. The K-S statistic of these distributions as a function of *θ* is presented. In the title we show the code of the individual and the value of *θ* that minimizes K-S, *θ**.

| name | Pareto (power law) | | | | Lognormal | | | | | Weibull | | | | | Gamma | | | | | θ^*^ |
| --- | --- | --- | --- | --- | --- | --- | --- | --- | --- | --- | --- | --- | --- | --- | --- | --- | --- | --- | --- | --- |
|  | α | K-S | Pval. | AIC_w_ | µ | Ơ | K-S | Pval. | AIC_w_ | a (scale) | b (shape) | K-S | Pval. | AIC_w_ | a (shape) | b (scale) | K-S | Pval. | AIC_w_ |  |
| 'f1' | 0.583 | 0.073 | 0.801 | 0.503 | -0.160 | 2.180 | 0.051 | 0.986 | 0.497 | 2.596 | 0.438 | 0.107 | 0.336 | 0.000 | 0.275 | 39.624 | 0.217 | 0.001 | 0.000 | 0.300 |
| 'f2' | 0.637 | 0.075 | 0.812 | 0.131 | 0.144 | 2.390 | 0.045 | 0.998 | 0.850 | 3.797 | 0.439 | 0.106 | 0.391 | 0.019 | 0.298 | 39.531 | 0.188 | 0.013 | 0.000 | 0.580 |
| 'g1' | 0.661 | 0.048 | 0.980 | 0.921 | -0.025 | 2.067 | 0.086 | 0.460 | 0.079 | 2.854 | 0.456 | 0.134 | 0.060 | 0.000 | 0.305 | 30.530 | 0.236 | 0.000 | 0.000 | 0.440 |
| 'h2' | 0.748 | 0.102 | 0.465 | 0.375 | -1.449 | 2.230 | 0.065 | 0.922 | 0.625 | 0.735 | 0.432 | 0.112 | 0.335 | 0.000 | 0.277 | 10.587 | 0.224 | 0.002 | 0.000 | 0.160 |
| 'i3' | 0.735 | 0.112 | 0.204 | 0.069 | -0.295 | 2.343 | 0.053 | 0.956 | 0.930 | 2.409 | 0.435 | 0.110 | 0.213 | 0.001 | 0.287 | 29.178 | 0.211 | 0.001 | 0.000 | 0.520 |
| 'i5' | 0.545 | 0.081 | 0.654 | 0.260 | -0.336 | 2.471 | 0.046 | 0.993 | 0.739 | 2.505 | 0.401 | 0.102 | 0.352 | 0.000 | 0.253 | 46.566 | 0.218 | 0.001 | 0.000 | 0.240 |
| 'j2' | 0.627 | 0.042 | 0.981 | 0.697 | -0.727 | 2.134 | 0.055 | 0.834 | 0.303 | 1.441 | 0.446 | 0.118 | 0.060 | 0.000 | 0.285 | 19.363 | 0.231 | 0.000 | 0.000 | 0.200 |
| 'f3' | 0.649 | 0.066 | 0.564 | 0.999 | -0.987 | 2.251 | 0.110 | 0.063 | 0.001 | 1.183 | 0.418 | 0.170 | 0.001 | 0.000 | 0.262 | 21.094 | 0.274 | 0.000 | 0.000 | 0.170 |
| 'f4' | 0.661 | 0.103 | 0.022 | 0.002 | -0.420 | 1.729 | 0.039 | 0.898 | 0.998 | 1.592 | 0.538 | 0.099 | 0.030 | 0.000 | 0.380 | 9.936 | 0.193 | 0.000 | 0.000 | 0.260 |
| 'f5' | 0.775 | 0.062 | 0.699 | 0.986 | -1.066 | 2.111 | 0.096 | 0.184 | 0.014 | 1.054 | 0.410 | 0.166 | 0.002 | 0.000 | 0.238 | 29.217 | 0.306 | 0.000 | 0.000 | 0.230 |
| 'g2' | 0.707 | 0.053 | 0.614 | 0.919 | -0.854 | 1.993 | 0.079 | 0.158 | 0.081 | 1.213 | 0.468 | 0.139 | 0.001 | 0.000 | 0.320 | 11.175 | 0.232 | 0.000 | 0.000 | 0.220 |
| 'j01' | 0.683 | 0.073 | 0.813 | 0.178 | -0.534 | 1.855 | 0.042 | 0.999 | 0.813 | 1.486 | 0.563 | 0.095 | 0.490 | 0.008 | 0.432 | 6.116 | 0.155 | 0.051 | 0.000 | 0.270 |
| 'k1' | 0.536 | 0.116 | 0.133 | 0.011 | -0.333 | 1.882 | 0.055 | 0.917 | 0.989 | 1.859 | 0.514 | 0.092 | 0.351 | 0.000 | 0.358 | 13.065 | 0.187 | 0.002 | 0.000 | 0.180 |
| 'k2' | 0.583 | 0.147 | 0.377 | 0.003 | -0.318 | 2.634 | 0.118 | 0.636 | 0.017 | 2.400 | 0.493 | 0.068 | 0.991 | 0.521 | 0.362 | 12.784 | 0.081 | 0.953 | 0.459 | 0.330 |
| 'l2' | 0.718 | 0.167 | 0.046 | 0.035 | -1.269 | 2.580 | 0.076 | 0.816 | 0.965 | 1.067 | 0.369 | 0.137 | 0.150 | 0.000 | 0.224 | 31.883 | 0.253 | 0.000 | 0.000 | 0.210 |
| 'm1' | 0.616 | 0.052 | 0.764 | 0.041 | -0.172 | 2.316 | 0.043 | 0.921 | 0.957 | 2.635 | 0.464 | 0.101 | 0.070 | 0.002 | 0.325 | 21.088 | 0.169 | 0.000 | 0.000 | 0.370 |
| 'm2' | 0.630 | 0.069 | 0.484 | 0.027 | -0.235 | 1.939 | 0.030 | 0.999 | 0.973 | 2.076 | 0.533 | 0.067 | 0.517 | 0.000 | 0.390 | 11.062 | 0.142 | 0.005 | 0.000 | 0.310 |
| 'm4' | 0.538 | 0.065 | 0.795 | 0.857 | -0.771 | 2.226 | 0.085 | 0.465 | 0.143 | 1.492 | 0.396 | 0.148 | 0.027 | 0.000 | 0.224 | 51.666 | 0.273 | 0.000 | 0.000 | 0.130 |
| 'n1' | 0.490 | 0.145 | 0.764 | 0.020 | 1.602 | 2.773 | 0.123 | 0.888 | 0.033 | 16.776 | 0.492 | 0.069 | 1.000 | 0.444 | 0.360 | 88.381 | 0.102 | 0.971 | 0.504 | 1.440 |
| n2' | 0.677 | 0.067 | 0.758 | 0.355 | -0.073 | 2.119 | 0.043 | 0.991 | 0.645 | 2.695 | 0.474 | 0.091 | 0.373 | 0.000 | 0.322 | 24.100 | 0.188 | 0.002 | 0.000 | 0.470 |

Table S1 – Results of the fits of stop duration distributions (in seconds) of 20 *A. boskianus* lizards. For each individual, we present the crossover that was found between long and short stops, *θ^*^*, as well as the properties of the fits of stops longer than *θ^*^* to four candidate distributions – Pareto, Lognormal, Weibull and Gamma. For each distribution we present the fitted parameters, the Kolmogorov-Smirnoff statistic (K-S) and its corresponding P-value, as well as the Akaike weight when the four different possible distributions are considered. The parameters of the Lognormal are the mean and SD of the corresponding normal distribution.
